# Lemur species-specific metapopulation responses to habitat loss and fragmentation

**DOI:** 10.1371/journal.pone.0195791

**Published:** 2018-05-09

**Authors:** Travis S. Steffens, Shawn M. Lehman

**Affiliations:** 1 Planet Madagascar, Toronto, Ontario, Canada; 2 Department of Anthropology, University of Toronto, Toronto, Ontario, Canada; Tierarztliche Hochschule Hannover, GERMANY

## Abstract

Determining what factors affect species occurrence is vital to the study of primate biogeography. We investigated the metapopulation dynamics of a lemur community consisting of eight species (*Avahi occidentalis*, *Propithecus coquereli*, *Microcebus murinus*, *Microcebus ravelobensis*, *Lepilemur edwardsi*, *Cheirogaleus medius*, *Eulemur mongoz*, and *Eulemur fulvus*) within fragmented tropical dry deciduous forest habitat in Ankarafantsika National Park, Madagascar. We measured fragment size and isolation of 42 fragments of forest ranging in size from 0.23 to 117.7 ha adjacent to continuous forest. Between June and November 2011, we conducted 1218 surveys and observed six of eight lemur species (*M*. *murinus*, *M*. *ravelobensis*, *C*. *medius*, *E*. *fulvus*, *P*. *coquereli*, and *L*. *edwardsi*) in the 42 fragments. We applied among patch incidence function models (IFMs) with various measures of dispersal and a mainland-island IFM to lemur species occurrence, with the aim of answering the following questions: 1) Do lemur species in dry deciduous forest fragments form metapopulations? 2) What are the separate effects of area (extinction risk) and connectivity/isolation (colonization potential) within a lemur metapopulation? 3) Within simulated metapopulations over time, how do area and connectivity/isolation affect occurrence? and 4) What are the conservation implications of our findings? We found that *M*. murinus formed either a mainland-island or an among patch metapopulation, *M*. *ravelobensis* formed a mainland-island metapopulation, *C*. *medius* and *E*. *fulvus* formed among patch metapopulations, and neither *P*. *coquereli* or *L*. *edwardsi* formed a metapopulation. Metapopulation dynamics and simulations suggest that area was a more consistent positive factor determining lemur species occurrence than fragment isolation and is crucial to the maintenance of lemur populations within this fragmented landscape. Using a metapopulation approach to lemur biogeography is critical for understanding how lemur species respond to forest loss and fragmentation.

## Introduction

Endemic to Madagascar, lemurs are the most endangered mammal group in the world, 94% of lemur species are threatened with extinction [1] largely due to habitat loss and fragmentation of the forests in Madagascar [2]. While there is some disagreement on precisely how much forest has been lost in Madagascar [3], researchers estimate that between 40 and 52 percent of the forest cover has been converted to non-forested habitat between the 1950s and 2010 [2–4]. The processes of habitat loss and fragmentation create landscapes with discrete fragments of habitat [5]. In western Madagascar, the forest is mostly rare tropical dry deciduous forest, and Ankarafantsika National Park contains one of the largest remaining intact portions of continuous dry forest. Dry forest is extremely sensitive to fire [6], which has resulted in a high degree of forest loss and increased habitat fragmentation in this area [4]. Indeed, satellite imagery shows that habitat loss and fragmentation continue in these tracks of continuous forest [7]. Therefore, even in protected areas such as Ankarafantsika, lemurs could be subject to increased habitat fragmentation.

The effects of forest loss and fragmentation on primate species occurrence are well studied [8–14]. Habitat loss is simply the removal of habitat from a landscape and habitat fragmentation is the separation of habitat into smaller less connected portions [5,15]. Typically, primate species occurrence decreases with increased forest loss [10,13,16–19]. Conversely, landscape connectivity/isolation appears to have little to no effect on individual primate occurrence when compared to fragment area [13,20–23]. The effect of habitat fragmentation separate from habitat loss on primate occurrence is not well understood [24–26]. To better understand how fragmentation impacts primate species, researchers need to further assess how connectivity, independent of habitat loss, affects primate occurrence [26].

Metapopulation dynamics offers multiple models for determining the population viability of lemur species in remnant forest patches. There are different types of single species metapopulation models with variable characteristics, including but not limited to the mainland-island metapopulation [27] and an among patch metapopulation model where colonization is not influenced by a mainland. A mainland-island metapopulation occurs where a patch or population within a fragmented landscape is particularly large (mainland) and is surrounded by smaller patches. The mainland has a large population of individuals that is unlikely to become extinct [28]. However, extinction risk is confounded by patch size [29], with smaller patches having a relatively higher extinction risk than larger patches. Because of its large size the mainland produces an unlimited supply of migrants called propagule rain (Hanski, 1994a). The mainland’s unlimited supply of migrants is independent of the number of patches occupied within the system [27]. The colonization potential or isolation of island patches is related to their distance from the mainland [27]. A mainland-island metapopulation may help explain the source-sink dynamics observed in some metapopulations [30]. Similar to a mainland-island model, in an among patch model extinction probability of a patch is a function of the area of that patch [29]. However, colonization is a negative exponential function of the distance to the nearest occupied patch plus a species-specific dispersal parameter [29].

Researchers have used metapopulation theory as a conservation tool to predict species persistence in a fragmented landscape under varying conservation strategies [8,31], to assess extinction risk [32], to determine factors impacting species occurrence [22], to determine species minimal critical forest patch size [22], and for population viability analyses [33]. Other studies have investigated the impact of fragment size and isolation within suspected metapopulations but they did not use metapopulation dynamics *per se* [9]. Despite metapopulation dynamics being widely recognized as a useful approach to determine how individual species respond to habitat loss and fragmentation [27,34,35], there is no research on metapopulation dynamics in lemurs and few studies on primates [8,22,33]. In one example of a study on primates, Chapman et al. [12] examined forest fragments along the periphery of Kibale National Park, Uganda and fitted a mainland-island incidence function model to occurrence data on four primate species. The metapopulation models accounted for a substantial amount of variation in each species occurrence. However, the authors found low confidence in the estimated coefficients for the models. For both *Procolobus badius* (red colobus) and *Colobus guereza* (black and white colobus), Chapman et al. [8] found a strong area effect on occurrence but little influence of connectivity on each species occurrence. For *Cercopithecus ascanius* (red-tailed monkey) fragment size or distance did not affect occurrence while Chapman et al. [8] found *Pan troglodytes* (chimpanzees) were an unsuitable species for the application of metapopulation dynamics because of their highly mobile nature. In another example of a metapopulation study on primates, Lawes et al. [11] examined a fragmented portion of *Podocarpus* forest in KwaZulu-Natal Province, South Africa, and applied a mainland-island incidence function model to *Cercopithecus mitis labiatus* occurrence and additional land use and environmental factors. Lawes et al. [22] found that the best-fit model incorporated only area as a factor determining *C*. *m*. *labiatus* occurrence. Model fit was not improved by the inclusion of isolation, land use, or other environmental factors.

Preliminary biogeography research on lemur species indicates considerable differences in lemur responses between continuous and fragmented forests [10,36–38]. For example, Steffens and Lehman [37] found that contrary to a previous study of two species of mouse lemurs (*Microcebus murinus* and *M*. *ravelobensis*) in continuous forest [39], there were significant, positive correlations between density and abundance for both species in forest fragments. Knowing that there are differences in biogeographic patterns in some species in continuous versus fragmented habitat raises the question: How will other species will respond to increased habitat fragmentation? Thus, understanding spatial variations in lemur responses to forest fragmentation is critical to a more informed understanding of their conservation biogeography.

The goal of this study is to investigate the vulnerability of eight lemur species to habitat loss and fragmentation in a fragmented landscape in Ankarafantsika National Park. Vulnerability was determined using different stochastic patch occupancy models (incidence function models (IFM)). Following to previous research on mammal patch occupancy in tropical environments [40], we do not employ standard hypothesis testing. Rather, we compare IFMs to answer the following questions: 1) Do lemur species in dry deciduous forest fragments form metapopulations? 2) What are the relative effects of area (extinction risk) and connectivity/isolation (colonization potential) within a lemur metapopulation? 3) Within simulated metapopulations over time, how do area and connectivity/isolation affect occurrence? and 4) What are the conservation implications of our findings?

## Methods

### Study site and study species

**Fig 1:**
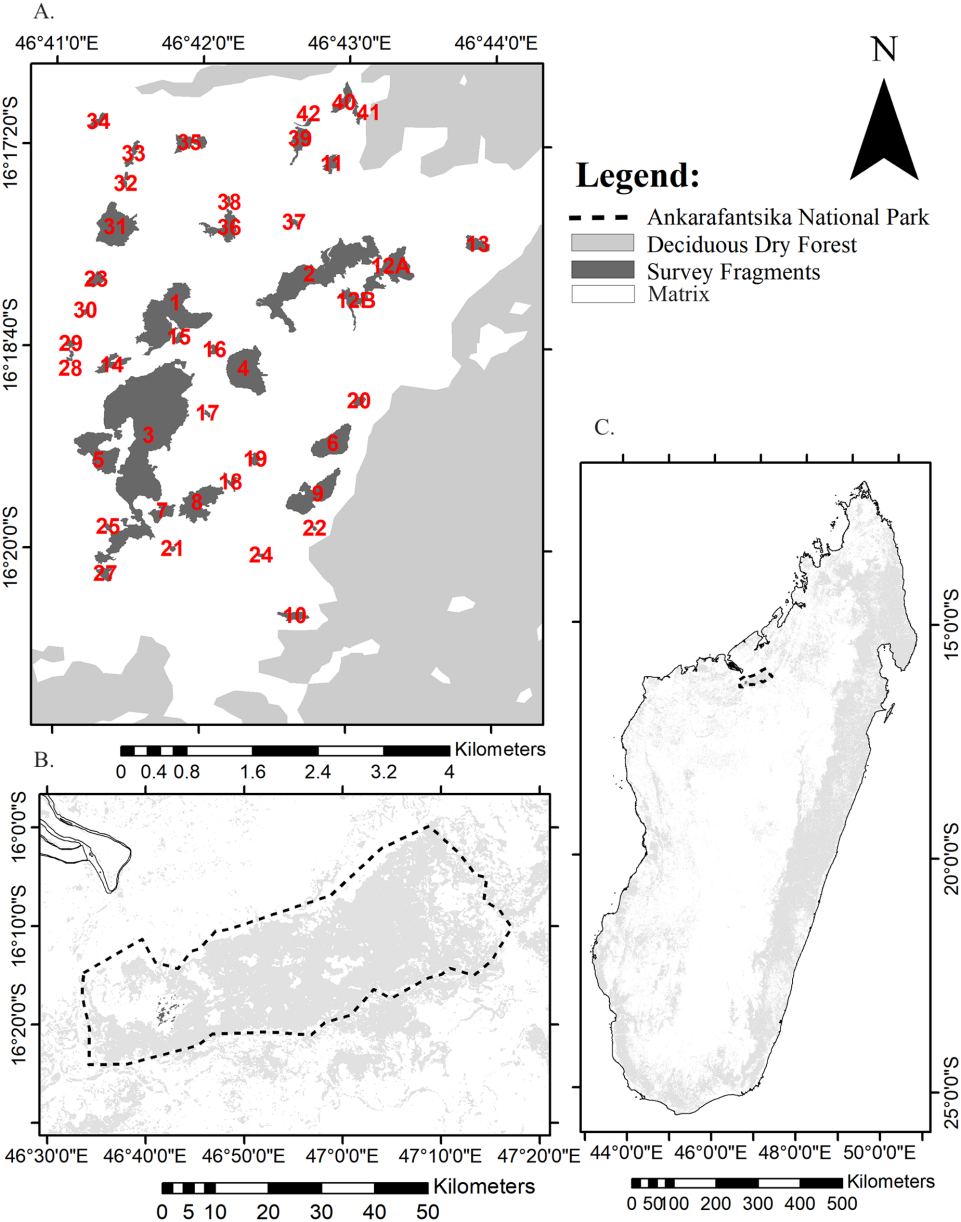
Study site and distribution of forest within Madagascar. a) Location of study site within Madagascar. b) Location of the study site within Ankarafantsika National park. c) Close up of study site showing the fragmented landscape, consisting of 42 fragments of dry deciduous forest separated by a mainly homogeneous matrix of grassland. Survey fragments are represented in dark grey and continuous forest in light grey and grassland in white.

We conducted this study in an approximately 3000 ha fragmented landscape consisting of 42 relatively homogeneous forest fragments surrounded by a relatively homogeneous grassland matrix within the western boundary of Ankarafantsika National Park (ANP), Madagascar (Fig 1). To the north, east, and south of the fragments there is continuous forest (Fig 1). We received permission to conduct our study from the Ministère de l'Environnement, de l'Ecologie et des Forêts, and Madagascar National Parks (Permit Number: 089/11/MEF/SG/DGF/DCB.SAP/SCB). ANP is approximately 135,800 ha, and consists of a mosaic of approximately 72,670 ha of dry deciduous forest and grassland [41,42]. The climate is mostly dry with mean yearly rainfall of 1,000–1,500 mm occurring mostly in the rainy season between November and April [41]. There are eight species of lemurs in ANP (Table 1). We conducted this study in the dry season and early part of the wet season (June–November) of 2011 to facilitate access, and because there is increased visibility due to reduced foliage. All species were active during the entire study except *C*. *medius*, which is in torpor between April and October [43].

**Table 1:** Primate species characteristics and patch occupancy in Ankarafantsika National Park found within study site.

Species	Body mass (g)	Activity pattern	Diet	Mean home range (ha)	Median dispersal distance (m) based on home range*	Median/Mean dispersal distance reported in literature (m)	IUCN status	# of occupied patches
*Cheirogaleus medius*	120–270	Nocturnal	Frugivore	1.55 ±0.42 [45]	873	N/A	Least Concern	12
*Microcebus murinus*	58–67	Nocturnal	Omnivore	2.83 ±1.44 [46]	1177	Median = 251 [44]	Least Concern	35
*Microcebus ravelobensis*	56–87	Nocturnal	Omnivore	0.59 ±0.11 [47]	538	Mean = 54 [45]	Endangered	34
*Propithecus coquereli*	3700–4300	Diurnal	Folivore	19.36 [48]	3080	N/A	Endangered	3
*Eulemur fulvus*	1700–2100	Cathemeral	Frugivore	13.5 [49]	2572	N/A	Near Threatened	7
*Eulemur mongoz*	1100–1600	Cathemeral	Frugivore	2.85 [50]	1182	N/A	Critically Endangered	0
*Lepilemur edwardsi*	1100	Nocturnal	Folivore	1.09 [51]	731	N/A	Endangered	2
*Avahi occidentalis*	800–1100	Nocturnal	Folivore	1.64 [51]	896	N/A	Endangered	0

*Median-dispersal distance was calculated as seven times the square root of the mean reported home range from each study.

To determine patch occupancy we used a single season visual survey along a single line transect within each of the 42 habitat fragments. We placed one survey transect along the longest axis of each fragment while going through the center of the fragment except in Fragment 12A where we placed the transect along the longest axis of the largest portion of the fragment, and fragment three where we had two transects. During survey walks each researcher walked slowly (approximately one km/hour), scanning and listening for all lemur species. During diurnal surveys one or two researchers scanned both sides of the transect simultaneously. Two researchers walked together during all nocturnal surveys and each researcher focused on one side of the transect for the entire duration of the survey. Each team member used high-powered flashlights and headlamps during nocturnal surveys to observe eye shine. Each team member carried binoculars to facilitate species identification and a laser range finder to measure distance metrics (below). We conducted diurnal and nocturnal surveys as follows: early diurnal surveys between 06:19 and 09:07 hours, late diurnal surveys between 14:39 and17:18 hours, early nocturnal surveys between 18:00 and 21:27 hours, and late nocturnal surveys between 02:17 and 5:55 hours. To ensure temporal independence for each survey, we only conducted one of each survey type (diurnal and nocturnal) per 24-hour period in each transect. To ensure spatial independence we alternated the direction of each transect walk. We surveyed all fragments at least twice during early June and between October and November to ensure an accurate assessment of the occurrence of *C*. *medius*, who can be in torpor between April and October [43,44]. In total, we conducted between 11 and 18 diurnal, and 11 and 21 nocturnal surveys in each fragment (S1 Table). Prior to the surveys, we trained the core team members on identifying each species within Ampijoroa field station. When we conducted surveys we ensured that there was at least one core team member experienced in identifying each of the eight different species on the survey. When team members observed a group or individual lemur, the team spent up to 15 minutes measuring and recording the following information: observer to animal distance, perpendicular distance of animal to transect, GPS location of the observer, angle of animal from transect, time, date, researchers names, which side the animal was detected, transect number, walk number, height animal was found, tree height animal was found, animal activity, group size, group spread, and species identity. Each of the species was easily identified by size, except for the two *Microcebus* species. These cryptic species are difficult to visually identify. Therefore, we used a suite of characteristics to determine the species identity of the two *Microcebus* species. We determined a positive identification of *M*. *murinus* only when the team observed all of the following characteristics: grey/brown fur, small body size, and a short tail that was thick at the base. We determined a positive identification of *M*. *ravelobensis* when the team observed all of the following characteristics: rufus fur, and a long tail that was thin at the base. We found it difficult to identify 41% of *Microcebus* sightings to the species level during surveys. Because of the cryptic nature of the *Microcebus spp*., in this study we conducted analysis on the sightings for *M*. *murinus* and *M*. *ravelobensis* when we were confident of their identification and second analysis combining all *Microcebus spp*. sightings. If we did not observe a species during any survey of a fragment, we considered it absent for that fragment.

### Question 1: Do lemur species in dry deciduous forest fragments form metapopulations?

In an incidence function model (IFM), incidence is a measure of the probability of species occurrence within a patch and is a function of both patch extinction probability and colonization potential [34]. It is difficult to measure patch extinction probability and colonization potential directly [34]. To determine a species extinction probability within a patch or patch network, it is necessary to acquire long-term data on mortality of individual primates, who are long lived, within patches of varying sizes. To determine colonization potential of a patch or patch network, it is necessary to know a species dispersal abilities between patches over more than one year. For primates this requires difficult to acquire long-term data on species dispersal patterns.

Using an IFM researchers can determine incidence in a metapopulation model without data on species extinction or colonization rates. From an IFM, we can infer the extinction probability of a patch and its colonization potential using simple occurrence data (presence/absence) gathered from a single-survey period among patches within a fragmented landscape [27,29]. An IFM uses area as a proxy for extinction risk and isolation as a proxy for colonization potential. Thus, it describes the probability that a species occurs (incidence) within a patch as a function of both the area (extinction risk) and isolation (colonization potential) of that patch [27,29]. The only additional data required are the sizes and locations of each patch and knowledge of the median-dispersal range of a species within the landscape. The benefit of an IFM is that it is more realistic because it incorporates patch area and isolation directly measured from the landscape and it can be easily parameterized based on occurrence data of species within a fragmented landscape at one particular point in time.

The incidence function models we used are spatially explicit models that have some simplifying assumptions including the following: that the patch has a size but no shape, the quality of the patch is constant, and the matrix is relatively uniform. The data needed for a metapopulation IFM include at least a single survey of patch occupancy within a network of patches, the x and y coordinates of each patch to determine the distance between each patch, patch area, and the species-specific dispersal ability within the landscape [27]. We selected our study site because it suits many assumptions of the model including having mostly homogeneous forest fragments of varying size separated by mostly homogeneous matrix of grassland. It is relatively easy to gather occurrence data for primates and to measure patch area and distances using current GPS and GIS technology. However, it is very difficult to know the dispersal ability of primates within a landscape.

We chose to investigate three different models including: 1. an among-patch incidence function model with rescue effect (including four associated sub-models: IFM, IFMproxy, IFMproxy2, IFMlit), 2. a mainland-island incidence function model without rescue effect (MI-IFM), and 3. a null model where lemur species occurrence varies randomly with respect to patch area and isolation (Null). The four among-patch sub-models are similar but differ in the way the dispersal parameter (*α*) was defined (see below).

For each model, we input data on patch occupancy for each species and *Microcebus spp*. combined, a measure of species-specific dispersal distance (*α*), patch area, and isolation (see below). We then took a linearized version of the IFM sub-models (see Eq (9) below) and the MI-IFM model (see Eq (15) below) and ran binomial generalized linear models (GLM) with a logit link function using the glm function in R [52], for each species and *Microcebus spp*. combined (adapted from [53]). For each GLM we ran we input species occurrence as the response variable and species-specific dispersal distance (*α*), patch area, and isolation as the predictor variables.

The IFM sub-models with rescue effect takes the following form [29,53]:

$$J_{i}=\frac{C_{i}}{C_{i}+E_{i}-C_{i}E_{i}}$$ (1)

$$E_{i}=\frac{e}{A_{i}^{x}},\;\text{for}\;A\geq e^{\frac{1}{x}}$$ (2)

$$M_{i}=\beta S=\beta \sum_{j\neq i}^{R}\text{exp}(-\alpha d_{ij})p_{j}A_{j}$$ (3)

$$C_{i}=\frac{M_{i}^{2}}{M_{i}^{2}+y^{2}}=\frac{S_{i}^{2}}{S_{i}^{2}+y},\;\text{where}\;y\;\text{absorbs}\;\beta$$ (4)

$$J_{i}=\frac{S_{i}^{2}A_{i}^{x}}{S_{i}^{2}A_{i}^{x}+ey}=\frac{1}{1+\frac{ey}{S_{i}^{2}A_{i}^{x}}}={\left[1+\frac{ey}{S_{i}^{2}A_{i}^{x}}\right]}^{-1}$$ (5)

where *J*_*i*_ in Eq (1) is patch incidence in patch *i* defined in terms of extinction and colonization rates, *E*_*i*_ is the extinction probability, *C*_*i*_ is the colonization probability, *S*_*i*_ is a measure of connectivity for patch i, and *A*_*i*_ is the area of patch *i*. *M*_*i*_ is a measure of connectivity within a landscape. J*i* in Eq (5) is patch incidence in patch *i* defined in terms of patch size *A*_*i*_ and patch connectivity *S*_*i*_. It is difficult to estimate extinction and colonization directly [27,29], however, it is possible to calculate *e* and *y* (parameters estimated from the data that relate to extinction and colonization probabilities respectively; see Question 2: What are the Separate Effects of Area (Extinction Risk) and Connectivity/Isolation (Colonization Potential) within a Lemur Metapopulation? for more explanation) with data on patch occupancy *p*_*j*_, patch size *A*, and connectivity *S* collected during a single time period survey [53]. Connectivity is estimated using the following:

$$S_{i}=\sum_{j\neq i}^{R}\text{exp}(-\alpha d_{ij})p_{j}A_{j}$$ (6)

where is *α* inverse of the median species-specific dispersal distance and *d*_*ij*_ is a distance matrix among patches. It is possible to change Eq (5) by applying a linear model for the log-odds of incidence:

$$J_{i}{\left[1+\frac{ey}{S_{i}^{2}A_{i}^{x}}\right]}^{-1}=\frac{1}{1+\text{exp}\left(\text{log}(ey)-2\text{log}(S_{i})-x\text{log}(A_{i})\right)}$$ (7)

$$\text{log}\left(\frac{J_{i}}{1-J_{i}}\right)=-\text{log}(ey)+2\text{log}(S_{i})+x\text{log}(A_{i})$$ (8)

A logit transformation results in:

$$logit(J_{i})=\beta_{0}+2\text{log}S+\beta_{1}x\text{log}A$$ (9)

The mainland-island incidence function model without rescue effect takes the following form (MI-IFM; [29]):

$$J_{i}=\frac{C_{i}}{C_{i}+E_{i}}$$ (10)

$$C_{i}=q\text{exp}(-\beta D_{i})$$ (11)

where *q* and *ß* are two parameters and *D*_*i*_ is the distance from the mainland to each island. Assuming that all the species are common on the mainland, where *C*_*i*_ approaches one when *D*_*i*_ approaches zero than *q* = 1 and Eq (11) can be simplified further [29]:

$$E_{i}=\frac{e}{A_{i}^{x}},\;\text{for}\;A\geq e^{\frac{1}{x}}$$ (12)

$$J_{i}={\left(1+\frac{\mu^{\beta D_{i}}}{A_{i}^{x}}\right)}^{-1}$$ (13)

It is possible to linearize Eq (13) by applying the log-odds of incidence:

$$\text{log}(J_{i})=-\text{log}(\mu )-\beta D_{i}+\beta_{2}\text{log}(A_{i})$$ (14)

A logit transformation then results in:

$$logit(J_{i})=\beta_{o}-\beta_{1}D+\beta_{2}\text{log}(A)$$ (15)

#### Patch occupancy

We determined patch occupancy of each fragment using the methods described above. We considered a species as present if we visually or acoustically (one instance) recorded their presence within a fragment. We considered a species as absent if we did not visually observe or acoustically confirm their presence within a fragment.

#### Dispersal distance

To determine connectivity within each IFM sub-model, we needed to determine the dispersal parameter (*α*). However, there is limited data on dispersal ability in most primates, especially lemurs. For the species in this study, dispersal distance has only been estimated only in *M*. *murinus* [54,55] and to a lesser degree of accuracy in *M*. *ravelobensis* [56]. Therefore, we ran multiple IFMs incorporating different dispersal parameters (*α*). For each species, we ran three IFM sub-models using different measures for dispersal, except for *M*. *murinus* and *M*. *ravelobensis* for which we ran four IFM sub-models with different measures for dispersal. For the first IFM sub-model, we determined which α fit the survey data by running all possible values for α and selecting the one that provided the lowest deviance (IFM; [53]). For the second IFM sub-model, we used a proxy for median-dispersal distance based on a function of the home range size of each species (IFMproxy) [57]. Bowman et al. [57] argues that median-dispersal distance could be estimated as the linear dimension (square root) of the mean home range multiplied by a factor of 7. Therefore, we took the mean reported home range for each species and determined its dispersal ability with the following formula:

$$\sqrt{home\;range\;(km^{2})}\times 7=median\;dispersal\;distance\;(km)$$ (16)

Alpha (*α*) is calculated as:

$$\frac{1}{median\;dispersal\;distance}$$ (17)

The proxy for dispersal using the formula from Bowman et al. [57] overestimated the median-dispersal of the two known species (*M*. *murinus* and *M*. *ravelobensis*; Table 1). Therefore, for the third IFM sub-model (IFMproxy2), we created a second proxy where we took the linear dimension of the mean reported home range:

$$\sqrt{home\;range\;(km^{2})}$$ (18)

Because lemurs like many arboreal primates may be dispersal limited [58] the value derived from formula (16) may overestimate median-dispersal distance. The dispersal distance values derived using formula (18) better fit the known dispersal distances for *M*. *murinus* and *M*. *ravelobensis* (Table 1). For the final IFM sub-model (IFMlit: *M*. *murinus* and *M*. *ravelobensis* only), we used the largest median-dispersal distance reported for each species regardless of sex (*M*. *murinus* = 251 m [54]; *M*. *ravelobensis* = 54 m [56]. For *Microcebus spp*. combined we included the all the sub-models as above but used both proxy dispersal estimates for both species (i.e. IFMproxyMM, IFMproxyMR, IFMproxy2MM, and IFMproxy2MR).

#### Patch area and isolation

To measure the area (ha) of each fragment we first walked the perimeter of each fragment recording the track with a handheld global positioning device (Garmin GPS map 60csx). We input the track into QGIS (2012; n = 38). If obstructions prevented a complete walk of the fragments perimeter (n = 4; Fragments 37, 39, 40, 42), we traced the fragments perimeter from a high resolution DigitalGlobe^™^ satellite image, via Google Earth^™^ taken during the study (10/8/2011). We input each polygon in QGIS and used the field calculator tool to determine the area of these fragments. Because an IFM does not incorporate shape of a fragment we estimated the edge-to-edge distance between each fragment by first calculating the center-to-center distance between each fragment using the ArcGIS Spatial Join tool and subtracting that by the radius of each fragment pairing assuming a circular shape for each fragment.

#### Model comparison

To determine which model was the most likely among the models/sub-models we tested we calculated corrected Akaike's information criterion with a correction for finite sample sizes (AICc) and then calculated AIC weights (*wi*; [59]). We considered the model with the highest *wi* as the model with the highest likelihood of being selected among the models/sub-models we tested [59]. We considered models with AICc values within two of the model with the lowest AICc as potential candidate models.

### Question 2: What are the separate effects of area (extinction risk) and connectivity/isolation (colonization potential) within a lemur metapopulation?

To determine if the incidence probability was positively related to area and connectivity (*S*_*i*_) we ran univariate GLM analysis on each species incidence probability against area and connectivity for the candidate model with the lowest AICc selected in question 1. We determined the incidence probability for each patch (*J*_*i*_) based on among patch models (Eq 1) or the mainland-island model (Eq 10). However, to calculate incidence probability (*J*_*i*_), we needed to determine the extinction probability (*E*_*i*_) and colonization probability (*C*_*i*_) for each patch. For the among patch models, with known patch sizes, patch occupancies, and connectivity, we used a generalized linear binomial model with logit link function (Eq (9)) to determine the coefficients *ß*_*0*_ and *ß*_*1*_ = *x* and to separate *e* (a parameter related to extinction probabilities) from *y* (a parameter related to colonization probabilities) to calculate the extinction and colonization probabilities for each patch using the following steps:

$$\hat{ey}=\text{exp}(-{\hat{\beta }}_{0})$$ (19)

$$\tilde{e}=\underset{p\neq 0}{\text{min}}A^{\hat{x}}$$ (20)

$$\tilde{y}=\hat{ey}/\tilde{e}$$ (21)

It is not possible to calculate *e* and *y* from the GLM directly (Eq 9) because any possible combinations of pairs of *e* and *y* giving the estimated $\hat{ey}$ are equally good [53]. Therefore following [53], we separated *e* and *y* from $\hat{ey}$ by fixing *e* as the smallest patch where a species was present as the area that extinction probability equals 1 (Eq 20) and dividing to determine *y* (Eq 21). We calculated the extinction probability (*E*_*i*_) and colonization probability (*C*_*i*_) and subsequently incidence probability (*J*_*i*_)of each patch using Eqs (2) and (4) respectively.

For the mainland-island model, we also used a generalized linear binomial model from a single survey of patch occupancy on Eq (15). Using this equation, we could determine the coefficients *ß*_*0*_ = μ, *ß*_*1*_ = *ß* and *ß*_*2*_ = *x* to calculate the colonization and extinction probabilities with Eqs (11) and (12) respectively. We then input the values from the colonization and extinction probabilities into Eq (10) to determine the incidence probability for each patch.

Using a Shapiro Wilk’s test we assessed normality for the following independent variables: area, species specific connectivity measures for the among patch models (*S*_*i*_), and the edge of fragment to continuous forest distance for the mainland-island models (DCF) [60]). We found that some variables needed transformation to meet the assumption of normality (e.g. log 10 for area, log 10 for *S*_*i*_ estimations for *E*. *fulvus*, and square root for the edge of fragment to continuous forest distance (DCF).

### Question 3: Within simulated metapopulations over time, how do area and connectivity/isolation affect occurrence? What are the conservation implications?

To see if there was a difference in how area affected occurrence compared to connectivity, we simulated metapopulation dynamics for each species over time based on the extinction and colonization probabilities derived from the IFM selected in question 1 in R following Oksanen [53]. We ran two sets of simulations. The first set represents a worst-case scenario where we ran a simulation separating out the five largest fragments and a second simulation where we separated out the five most connected/closest fragments. The second set represents the opposite scenario where fragments that are smaller and least connected/furthest were removed from the simulation. For each species and *Microcebus spp*. combined, we then ran the two sets of two simulations for 200 time steps (equivalent of 200 years). In the first half of the simulation all 42 fragments contribute to the metapopulation. At time step 101, we continue simulations on the five removed fragments (i.e. five largest, five smallest, five most connected, five least connected) and on the remaining 37 fragments for 99 more time steps. This method allows the ability to model what happens to lemur species occupancy in the five removed and remaining 37 fragments independently of each other. We used the number five because this represented a realistic conservation scenario where in a single event five of the largest, smallest, least/furthest, or most connected/closest fragments could be lost.

## Results

We sampled 42 fragments within the study landscape with a median size of 2.16 and range of 0.23–117.70 ha. The mean distance between centroids of each fragment was 2.82 ± 1.36 km with a range of 0.15 km to 6.48 km. The proxy for median-dispersal distance using Eq (16) ranged from 538 m (*M*. *ravelobensis*) to 3080 m (*P*. *coquereli*) and using Eq (18) ranged from 77 m (*M*. *ravelobensis*) and 400 m (*P*. *coquereli*: Table 1). Patch occupancy differed among species. Smaller-bodied *Cheirogaleids* occurred in the largest number of fragments while the remaining three larger species occurred in the fewest (Table 1). However, a linear regression of frequency of occupancy versus body size yielded no relationships (adjusted R^2^ = 0.33, P = 0.14). Transect and survey data are summarized in S1 Table. We did not observe any *A*. *occidentalis* or *E*. *mongoz* individuals during our study. Therefore, we did not include these species in the analysis.

### Question 1: Do lemur species in dry deciduous forest fragments form metapopulations?

**Fig 2:**
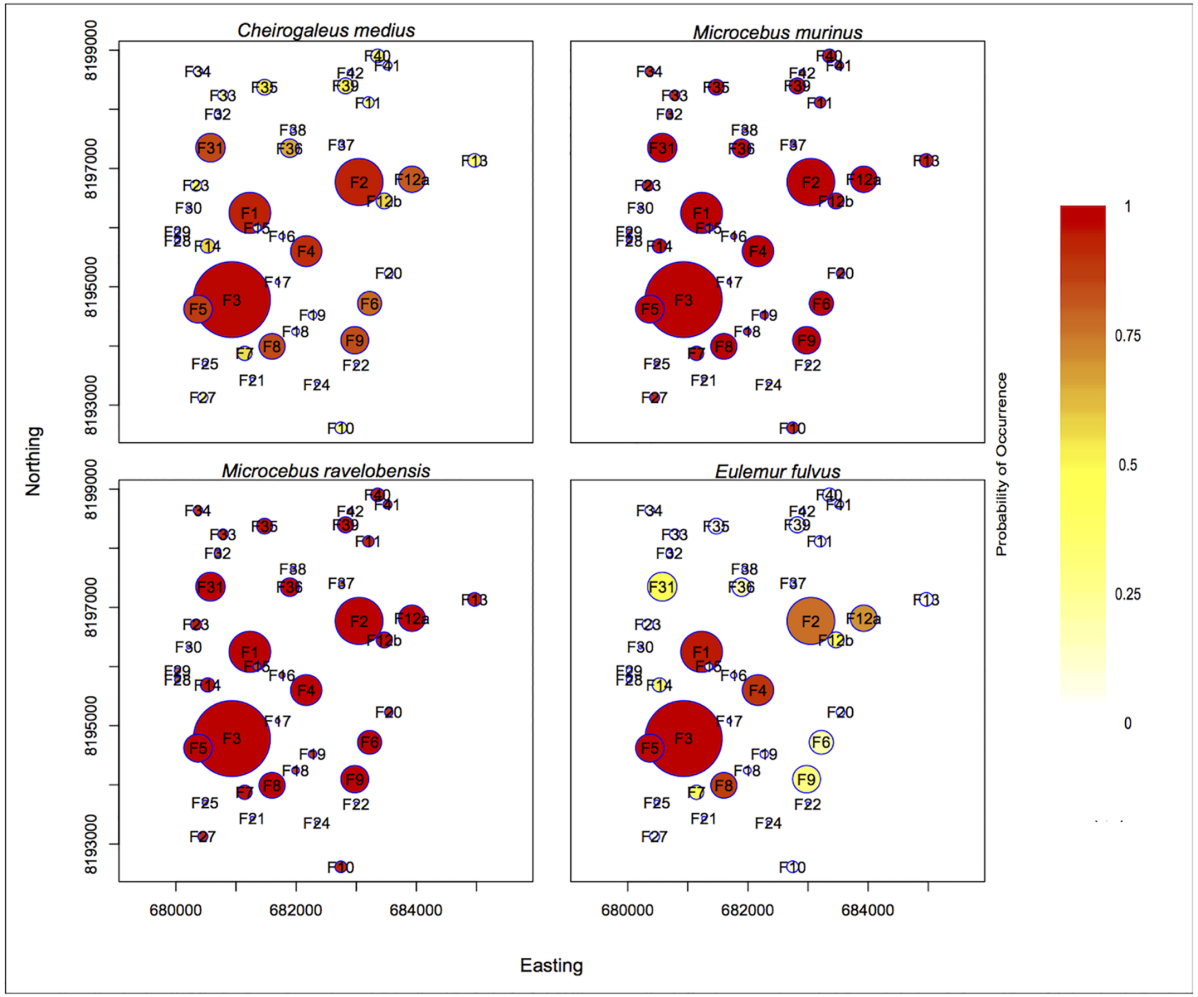
Probability of occurrence among patches for four lemur species in a fragmented landscape. Colors represent the probability of occurrence: red reflects the highest probability of occurrence for a species within a fragment and white the lowest. The probability of occurrence is based on the fitted incidence function model with α parameterized from the data (IFM) for *C*. *medius*, *M*. *murinus*, and *E*. *fulvus* and the IFM with α determined from the literature (IFMlit) for *M*. *ravelobensis*. The size of each circle represents the size of each fragment relative to one another. The position of fragments is based on Universal Transverse Mercator (UTM) coordinate system. Northing is equivalent to latitude and easting is equivalent to longitude.

The probability of occurrence differed among species (Fig 2). Both *Microcebus* species had the highest probability of occurrence in the landscape followed by *C*. *medius*. *E*. *fulvus* had the lowest probability of occurrence within the landscape.

We found differences in model selection results between species (Table 2). For *C*. *medius*, the among patch sub-model where dispersal was determined based on the IFM had the lowest AICc (IFM: Table 2). No other models had AICc values within two of the lowest model for *C*. *medius*. For both *M*. *murinus* and *M*. *ravelobensis* separately and combined the mainland-island model had the lowest AICc (Table 2). However, for *M*. *murinus* and both *Microcebus spp*. combined we found additional candidate models within two AICc values of the lowest model including the among patch (IFM) model and the null model (Table 2). These results suggest that when these species data are combined it is likely that they do not form either a mainland island or an among patch metapopulation (Table 2). For both *P*. *coquereli* and *L*. *edwardsi*, there were no models with values lower than or within two of the null model suggesting these species do not form either type of metapopulation that we tested for. However, for *P*. *coquereli* the among patch model was close at 2.31 AICc away from the null model (Table 2). For *E*. *fulvus*, the model with the lowest AICc value was IFM followed by IFMproxy as the only other model within two AICc values (Table 2). Therefore, we found support for among patch metapopulations in *C*. *medius* and *E*. *fulvus* and mainland-island metapopulations in *M*. *murinus* and *M*. *ravelobensis*. However, we found no support for the formation of metapopulations for *P*. *coquereli* or *L*. *edwardsi*. We found limited support for a mainland-island metapopulation when the *Microcebus* data was combined at the genus level.

**Table 2:** Metapopulation models of six lemur species in 42 fragments in a fragmented landscape.

Species	Model^a^	K	AICc	ΔAICc	*Wi*	Log Likelihood
*Cheirogaleus medius*	IFM	2	30.83	0	0.66	-13.26
	MI-IFM	3	33.12	2.29	0.21	-13.24
	IFMproxy	2	34.07	3.24	0.13	-14.88
	NULL	2	56.75	25.92	0	-26.22
	IFMproxy2	2	83.73	52.9	0	-39.71
*Microcebus murinus*	MI-IFM	3	31.88	0	0.72	-12.62
	IFM	2	33.81	1.93	0.27	-14.75
	NULL	2	40.59	8.71	0.01	-18.14
	IFMproxy	2	44.61	12.73	0	-20.15
	IFMlit	2	64.05	32.17	0	-29.87
	IFMproxy2	2	70	38.12	0	-32.85
*Microcebus ravelobensis*	MI-IFM	3	33.28	0	0.98	-13.32
	IFM	2	42.73	9.45	0.01	-19.21
	IFMproxy	2	43.59	10.32	0.01	-19.64
	NULL	2	44.98	11.7	0	-20.34
	IFMproxy2	2	76.35	43.07	0	-36.02
	IFMlit	2	111.51	78.23	0	-53.6
*Microcebus spp*. Combined	MI-IFM	3	20.19	0	0.33	-6.78
	NULL	2	20.54	0.35	0.28	-8.11
	IFM	2	20.69	0.51	0.26	-8.19
	IFMproxyMM	2	23.22	3.03	0.07	-9.45
	IFMproxy2M	2	23.43	3.24	0.07	-9.56
	IFMproxy2M	2	31.38	11.2	0	-13.54
	IFMproxyMR	2	31.38	11.2	0	-13.54
*Propithecus coquereli*	NULL	2	9.55	0	0.56	-2.62
	IFM	2	11.85	2.31	0.18	-3.77
	IFMproxy	2	11.98	2.43	0.17	-3.83
	MI-IFM	3	12.95	3.41	0.1	-3.16
	IFMproxy2	2	148.48	138.94	0	-72.09
*Eulemur fulvus*	IFM	2	9.64	0	0.5	-2.67
	IFMproxy2	2	9.7	0.06	0.49	-2.7
	IFMproxy	2	17.57	7.93	0.01	-6.63
	MI-IFM	3	19.53	9.89	0	-6.45
	NULL	2	40.59	30.95	0	-18.14
*Lepilemur edwardsi*	NULL	2	-6.42	0	1	5.36
	MI-IFM	3	12.8	19.22	0	-3.08
	IFM	2	19.09	25.51	0	-7.39
	IFMproxy	2	22.85	29.27	0	-9.27
	IFMproxy2	2	148.48	154.9	0	-72.09

^a^ IFM = incidence function model where *α* was parameterized based on occupancy data from one survey period; MI-IFM = mainland-island incidence function model; IFMproxy, IFMproxyMM, and IFMproxyMR = IFM where *α* was calculated as a proxy for dispersal ability based on the square root of the mean home range multiplied by seven reported for each species in the literature (MM represents *M*. *murinus* and MR represents *M*. *ravelobensis*); IFMproxy2, IFMproxy2MM, and IFMproxy2MR = IFM where *α* was calculated as a proxy for dispersal ability based on the square root of the mean home range reported for each species in the literature; IFMlit = IFM where *α* was based on the literature for species where data has been reported on dispersal ability. K = number of parameters in the model.

### Question 2: What are the separate effects of area (extinction risk) and connectivity/isolation (colonization potential) on a lemur metapopulation?

We found that log10 area was a significant positive contributor to incidence probability for all species for all models (Table 3). However, the results for connectivity (*S*_*i*_) were more complicated. For the among patch models, connectivity (*S*_*i*_) was not a significant contributor to incidence probability for *C*. *medius* (Table 3). The square root of connectivity (sqrt(*S*_*i*_)) was a significant positive contributor to incidence probability for sub-model IFM for *E*. *fulvus* (Table 3). For the mainland-island models, the square root of the distance to continuous forest (sqrt(DCF)) was a significant negative contributor to incidence for *M*. *murinus*, *M*. *ravelobensis*, and *Microcebus spp*. combined (Table 3).

**Table 3:** Univariate GLM results for the probability of occurrence (*J*_*i*_) for lemur species against area and connectivity.

Species	Metapopulation Model^a^	GLM Model	Coefficient Value	Estimate	Standard Error	t-value	p-value
*Cheirogaleus medius*	IFM	(***J***_***i***_) = B+Blog10Area	Intercept	0.9903	0.0578	17.1	<0.01
			log10 Area	0.4295	0.0339	12.5	<0.01
		(***J***_***i***_) = B+B*(*S*_*i*_)	Intercept	0.32088	0.27173	1.18	0.24
			*S*_*i*_	-0.0467	0.16724	-0.03	0.98
*Microcebus murinus*	MI-IFM	(***J***_***i***_) = B+Blog10Area	Intercept	1.2737	0.0682	18.69	<0.01
			log10 Area	0.3341	0.04	8.35	<0.01
		(***J***_***i***_) = B+B*sqrtDCF	Intercept	1.0755	0.0912	11.8	<0.01
			sqrt(DCF)	-0.3635	0.0925	-3.93	<0.01
*Microcebus ravelobensis*	IFMlit	(***J***_***i***_) = B+Blog10Area	Intercept	1.2583	0.0585	21.49	<0.01
			log10 Area	0.2884	0.0344	8.39	<0.01
		(***J***_***i***_) = B+B*sqrtDCF	Intercept	1.0474	0.0824	12.71	<0.01
			sqrt(DCF)	-0.2697	0.0863	-3.23	<0.01
*Microcebus spp*.	MI-IFM	(***J***_***i***_) = B+Blog10Area	Intercept	1.2394	0.0679	18.26	<0.01
			log10 Area	0.2307	0.0398	5.79	<0.01
		(***J***_***i***_) = B+B*sqrtDCF	Intercept	1.059	0.0816	12.98	<0.01
			sqrt(DCF)	-0.2028	0.0828	-2.45	0.02
*Eulemur fulvus*	IFM	(***J***_***i***_) = B+Blog10Area	Intercept	0.7493	0.0928	8.08	<0.01
			log10 Area	0.3694	0.0544	6.79	<0.01
		(***J***_***i***_) = B+B*sqrt(*S*_*i*_)	Intercept	0.4095	0.1166	3.51	<0.01
			sqrt(*S*_*i*_)	0.1206	0.0527	2.29	0.03

^a^ See Table 2 for definitions.

### Question 3: Within simulated metapopulations over time, how do area and connectivity/isolation affect occurrence?

**Fig 3:**
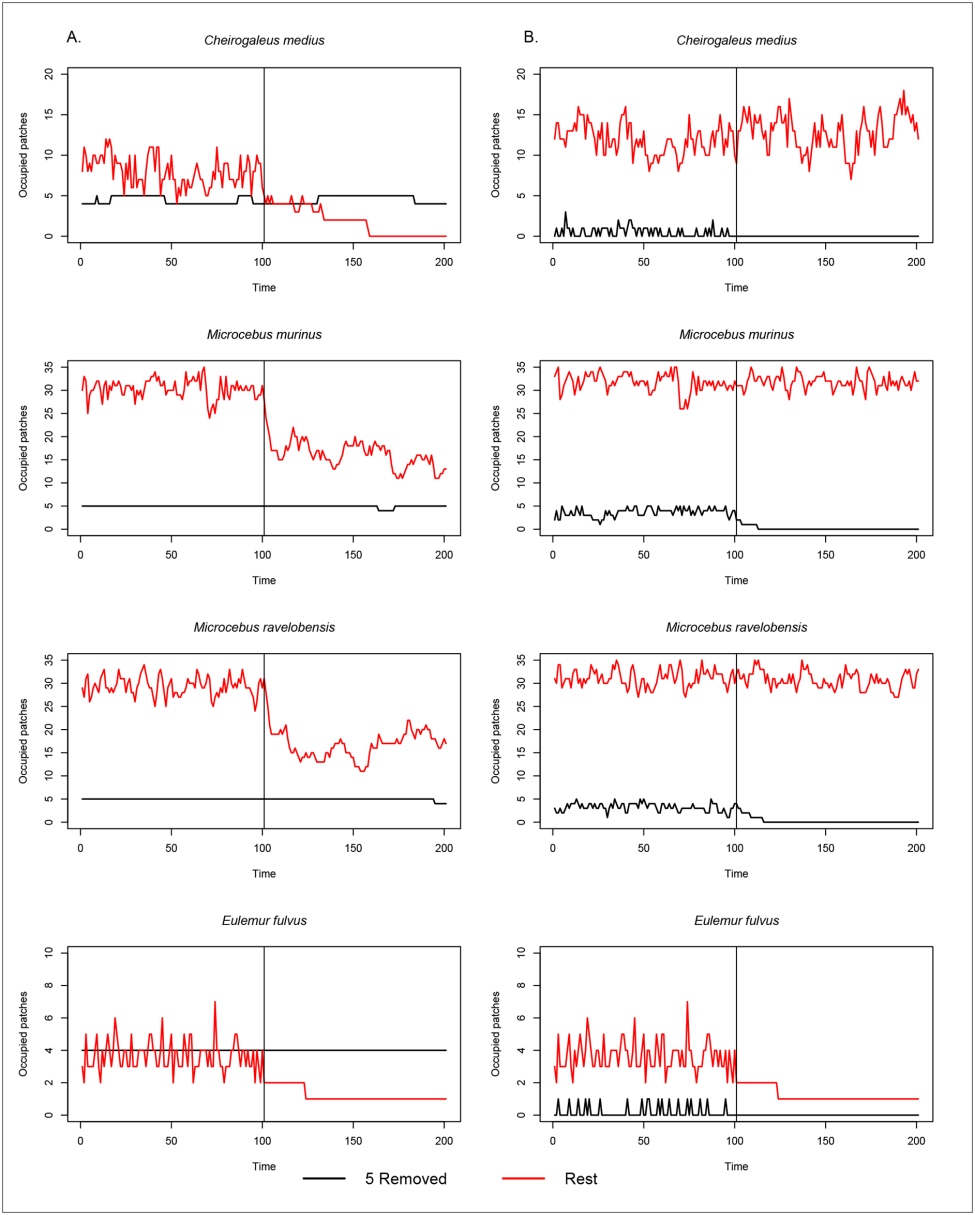
Simulations of metapopulation dynamics for four lemur species over 200 time steps in a fragmented landscape when the five largest and five smallest fragments are removed. Simulated species occurrence over time using a Markov chain process. The five largest (A) and five smallest (B) fragments (black lines), respectively are removed from the rest of the fragments (n = 37; red lines) at time period 101(vertical line). After this point we ran simulations, to time period 200, separately to demonstrate the impact of either removing the largest (A) or smallest (B) fragments (black). We ran simulations using the IFM with α parameterized from the data (IFM) for *C*. *medius*, *M*. *murinus*, and *E*. *fulvus* and the IFM with α determined from the literature (IFMlit) for *M*. *ravelobensis*.

**Fig 4:**
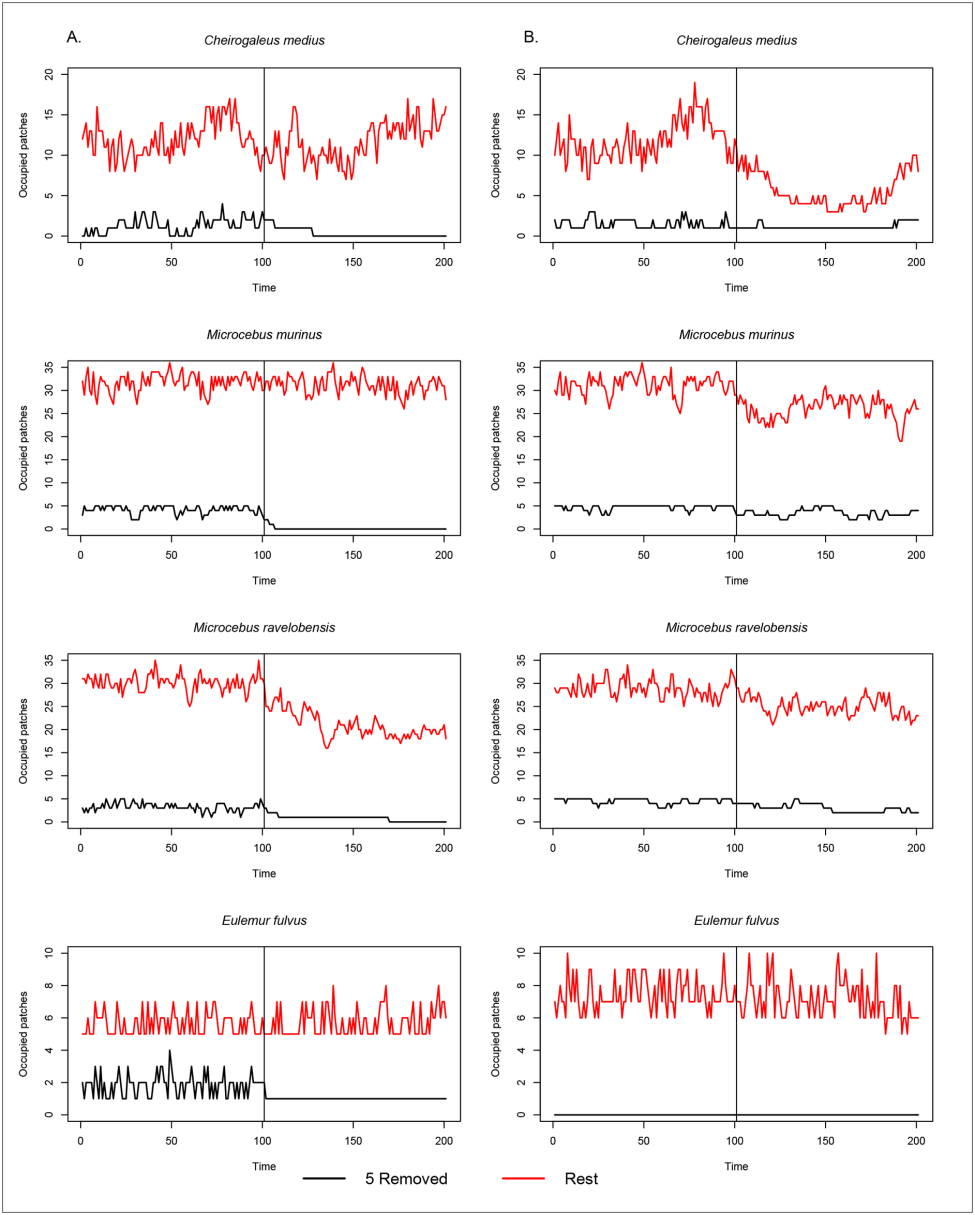
Simulations of metapopulation dynamics for four lemur species over 200 time steps in a fragmented landscape when the five most connected and five least connected fragments are removed. Simulated species occurrence over time using a Markov chain process. We removed the five most connected/closest (A) and five least connected/furthest (B) fragments, respectively (black lines) from the rest of the fragments (n = 37; red lines) at time period 101(vertical line). After this point we ran simulations, to time period 200, separately to demonstrate the impact of either removing the most/closest (A) or least/furthest (B) connected fragments (black). We ran simulations using the IFM with α parameterized from the data (IFM) for *C*. *medius*, *M*. *murinus*, and *E*. *fulvus* and the IFM with α determined from the literature (IFMlit) for *M*. *ravelobensis*.

Fragment area has a greater influence than fragment isolation on overall species occurrence. Removal of the five largest fragments via simulation caused all species to decline in occurrence (Fig 3A). The most extreme example was *C*. *medius*, which became extinct in the remaining fragments. None of the species showed any appreciable change in occurrence among the five largest fragments when they were separated from the remaining 37, although occurrence of *C*. *medius* did vary between three and five in the five largest fragments. Removing the five smallest fragments (Fig 3B) caused no noticeable decline in species occurrence in the 37 remaining fragments. However, separation of the five smallest fragments from the remaining 37 caused a decline in occurrence of all species in the five smallest fragments. Removal of the five most connected/closest fragments via simulation resulted in no obvious changes in occurrence for any species except *M*. *ravelobensis* which declined following the removal of the five closest fragments to the continuous forest (Fig 4A). Occurrence for all four species declined within the five most connected fragments following separation from the remaining 37 patches. Removal of the five least connected/furthest fragments in no change in occurrence for *E*. *fulvus* but caused a declining trend in occurrence for *C*. *medius* and to a lesser degree *M*. *murinus* followed by *M*. *ravelobensis* following removal of the five least connected fragments (Fig 4B). In the five least connected/furthest fragments there was little difference before and after separation from the remaining 37.

## Discussion

Using a single-season survey of patch occupancy, we found that six lemur species respond differently to area and connectivity/isolation in a fragmented landscape. Simulations of metapopulation dynamics provide support that lemur species occurrence was consistently positively related to area but had mixed results for connectivity. For both *Microcebus* species, occurrence was negatively related to connectivity, while *E*. *fulvus* occurrence was positively related to connectivity. Based on the simulation results, we suggest that the most connected fragments are not as important to the maintenance of the metapopulation as would be predicted by metapopulation theory [34]. For example, when we separated the most connected/closest fragments from the remaining fragments in the system only the simulated occurrence for *M*. *ravelobensis* declined within the most connected fragments. However, when we separated the least connected/furthest fragments our simulations showed declines in species occurrence for *C*. *medius*, *M*. *murinus*, and *M*. *ravelobensis*.

We would like to identify some potential caveats that could impact the interpretation of our results. The first is the known difficulty in visually assessing *Microcebus spp*. in our study area. We acknowledge this difficulty and warn readers to consider the implications of misidentification when interpreting the results. Our research teams are currently using mark-recapture methods combined with genetic analysis to determine if visual surveys provide an accurate representation of species determination in mouse lemurs. It is not only difficult to visually determine species identity but there is also the potential for hybridization between the two *Microcebus spp*. We observed numerous occurrences of individuals that had what appeared to be mixed characteristics. In total we were unsure in the identification of 41% of our *Microcebus spp*. sightings. Further genetic research is needed to determine if hybridization is occurring within the landscape. The second caveat is the fact that *C*. *medius* goes into torpor between April and October. In an attempt to determine the occurrence of *C*. *medius* we re-surveyed each fragment (two more times) where *C*. *medius* had not been observed when *C*. *medius* was coming out of torpor. We found that *C*. *medius* are extremely active and very conspicuous when coming out of torpor. However, it is important to note that the limited number of surveys we conducted when *C*. *medius* was potentially active may impact our results for this species.

### Question 1: Do lemur species in dry deciduous forest fragments form metapopulations?

Metapopulation dynamics represents an ideal approach for conservation biogeography under the following circumstances: when the habitat is in discrete patches, when ecological processes occur at the local and metapopulation scale, when habitat within the discrete spatial unit is large enough for local breeding populations, and when the patches are relatively permanent [34]. The level of habitat loss and fragmentation in Madagascar provides an ideal situation in which to apply a metapopulation approach to studying lemur populations: there is only a fraction of habitat remaining, forest patches (fragments) are discrete units separated by a non-habitat matrix, many patches are large enough to maintain local breeding populations, and local populations are connected to one another through dispersal, thus creating metapopulations.

We found support for only the null model (where occurrence was randomly related to patch area and connectivity) for *P*. *coquereli* and *L*. *edwardsi*, suggesting these species did not form metapopulations within our study site. Both of these species only occurred within a small number of large fragments within the landscape (three for *P*. *coquereli* and two for *L*. *edwardsi*) and appear to be more impacted by habitat loss and fragmentation than the other species we studied.

It is possible that the existing populations of the two species within the fragments declined and became locally extinct over time because fragment size was too small to support local populations. The majority of the fragments are smaller than home ranges reported for *P*. *coquereli* [48]. Warren and Crompton [51] found that for *L*. *edwardsi*, yearly home range size was 0.81–1.70 ha and Rasoloharijaona et al. [61] found median home range size for *L*. *edwardsi* ranged between 0.98 ha (females) and 1.0 ha (males). These home range sizes suggests that *L*. *edwardsi* should be able to tolerate smaller fragments, however their mean horizontal distance travelled per day was quite large. Warren and Crompton [51] found that *L*. *edwardsi* could travel as much as 463 m (horizontal travel distance) per day. Few fragments in our study site had linear dimensions greater than 463 m. *L*. *edwardsi* occurrence appears to differ in continuous versus fragmented habitats. For example, Craul et al. [10] found *L*. *edwardsi* to occur in 13 of 17 continuous forest sites surveyed but only two of six habitat fragments, a finding that supports the hypothesis that this species is not tolerant to habitat loss and fragmentation. We found both *P*. *coquereli* and *L*. *edwardsi* to occur only in larger fragments (the three largest for *P*. *coquereli* and the largest and fourth largest for *L*. *edwardsi*), and both were absent in all fragments smaller than 11.58 ha. Therefore, the explanation of fragments being too small for survival is plausible for both *P*. *coquereli* and *L*. *edwardsi*. *L*. *edwardsi* may also be particularly sensitive to anthropogenic disturbance. Rabesandratana et al. [62] undertook a survey of *L*. *edwardsi* at 10 sites in Ankarafantsika National Park, but not in the vicinity of our study. Although this lemur species was present at 9 of the 10 sites, density estimates were low for sites subject to anthropogenic disturbance (e.g., near villages and areas for palm exploitation). Thus, the proximity of our research site to local villages may indicate the deleterious effects of anthropogenics on *L*. *edwardsi*.

Minimum area requirements, limited numbers of large trees, and hunting pressure may also relate to the absence of *A*. *occidentalis* in the forest fragments. In eastern littoral forests, Norscia [63] only found *Avahi meridionalis* in forest fragments larger than 75 ha and no correlation between fragment area and *Avahi* density. Although *A*. *occidentalis* have small home ranges (median range between 1.57 ha– 1.79 ha, [64]) and rely on low quality abundant leaves, suggesting that they would be able to inhabit relatively small fragments, it is possible that the number and distribution of large trees impacts *Avahi* occurrence [63]. Norscia [63] found that the percentage of large trees above 3.2 cm DBH was significantly positively related to *Avahi* density. Illegal hunting does occur within the park, and the although the most commonly hunted species are the larger-bodied and more common *P*. *coquereli* and *E*. *fulvus*, García and Goodman [65] did identify remains of *A*. *occidentalis* from a hunt within the park. This lemur species is also thought to be particularly sensitive to seasonal burning of grasslands adjacent to forest fragments, which is undertaken by local people to promote fresh browse for domestic cattle.

Both *Microcebus spp*. appear to form mainland-island metapopulations. Dispersal ability differs between *M*. *murinus* and *M*. *ravelobensis* in continuous forest [39,54–56] with *M*. *ravelobensis* possibly more dispersal limited than *M*. *murinus* with estimated 0.05 km [56] and 0.25 km [54] median-dispersal distances respectively. We found that our estimates of dispersal ability determined from the metapopulation models were vastly higher than those reported for each species (10 km for *M*. *murinus* and 0.27 km for *M*. *ravelobensis*). However, the trend of M. murinus having a higher dispersal ability remained. It should be noted that dispersal derived from incidence function models can be inaccurate [53]. It appears that *M*. *ravelobensis* is more dispersal limited than *M*. *murinus* in both continuous and fragmented forest. If *M*. *ravelobensis* is more dispersal limited then why do both species exhibit similar patterns of occurrence within the landscape? *M*. *murinus* prefers higher elevation and drier forests than *M*. *ravelobensis* [39]. However, our study site was chosen for its relative homogeneity. In a study in the same landscape Steffens and Lehman [37] found that abundance in both *M*. *murinus* and *M*. *ravelobensis* were related to similar factors such as dendrometrics, fragment area, and isolation. In a continuous forest, Burke and Lehman [66] found differences between *M*. *murinus* and *M*. *ravelobensis* in the capture rates of each species and the body mass of female *M*. *ravelobensis* between the edge and interior habitat. They captured more *M*. *ravelobensis* and fewer *M*. *murinus* along the edge than in the interior habitat. They found female *M*. *ravelobensis* along the edge had greater body mass than those in the interior habitat. Therefore, both species may form mainland-island metapopulations but for different reasons. We suggest *M*. *murinus* is more capable of dispersing to further fragments than *M*. *ravelobensis*, but due to greater edge tolerance *M*. *ravelobensis* is more capable of surviving in fragments, at least in the short term. This would imply that *M*. *murinus* may be forming a source-sink metapopulation.

For both *C*. *medius* and *E*. *fulvus*, the most likely among patch incidence function sub-model were where we estimated the dispersal parameter (*α*) using the survey data (IFM). For *E*. *fulvus* the sub-model where dispersal was based on the linear dimension of their mean home range (IFMproxy2) was nearly as likely as the IFM model. Based on the support for these models and lack of support for the mainland-island model for both lemur species it appears they are sufficiently capable of moving between fragments to colonize extinct fragments without needing to rely on the mainland for more migrants. *C*. *medius* and *E*. *fulvus* were the only two frugivores in the study site. Larger-bodied species tend to have greater dispersal ability than smaller species [67] and frugivorous primates tend to have larger home ranges than folivorous primates [68,69]. Thus, home range size may predict dispersal ability [57] and frugivores have greater dispersal ability than folivores. However, *E*. *fulvus* occurred in fewer fragments than *C*. *medius*. *C*. *medius* with its small body mass and hibernation patterns, may be better suited to survive in more fragments than *E*. *fulvus*. For example, we found *C*. *medius* in fragments as small as 1.69 ha. Within this landscape there were likely few fragments large enough for *E*. *fulvus* to live in, which required *E*. *fulvus* to move between fragments. *E*. *fulvus* may be transient within patches that are smaller than they would normally need to be able to survive. For example, we found *E*. *fulvus* in one fragment that was smaller (4.16 ha) than their reported home range yet absent in four fragments that were within their reported home range. *E*. *fulvus* is smaller in body mass but occurs in larger groups than *P*. *coquereli* which is why they both have similar home range sizes (Table 1; [48,49]). However, *E*. *fulvus* occurred in seven fragments and *P*. *coquereli* only two fragments. *P*. *coquereli* may be limited by edge effects that reduce habitat suitability, hunting avoidance, or predator avoidance [48,70,71] where *E*. *fulvus* may be more edge tolerant. A study on *P*. *coquereli* distribution found that they are a capable of living in degraded habitat [72]. Further study is needed to determine why *P*. *coquereli* appears capable of living in degraded habitats but also appears edge avoidant. Regarding *E*. *fulvus* Lehman et al. [73,74] found that contrary to predictions *Eulemur rubriventer* was edge tolerant. Lehman [73] suggests that another species of *Eulemur*, *E*. *rubriventer*, behaved more like a folivore/frugivore than a strict folivore. It is possible that *E*. *fulvus* behaved the same way. However, we need further study to determine if *E*. *fulvus* is more folivorous or frugivorous within the fragments and to determine what is the availability of fruiting trees within the fragments.

Unlike any of the other species observed within the fragments, *C*. *medius* is capable of extended hibernation [43]. Like other *Cheirogaleus* species, *C*. *medius* consumes large amounts of high-sugar fruits prior to hibernation in order to build up fat reserves [44]. Therefore, *C*. *medius* may be able to survive only in fragments that have a high availability of fruit during this crucial period. Tree holes used by *C*. *medius* must be carefully selected in order to allow sufficient maintenance of body temperature during the months that they hibernate [43]. Like fruit availability, tree holes may be a limiting resource for *C*. *medius* in the fragments.

### Question 2: What are the separate effects of area (extinction risk) and connectivity/isolation (colonization potential) on a lemur metapopulation?

#### Area

We found that fragment area was positively related to each species probability of occurrence, regardless of the selected candidate model. In metapopulation dynamics as local populations grow and reach their carrying capacity, individuals are forced to leave the patch to find a new suitable patch [75]. An empty patch is considered colonized when an immigrant arrives and subsequently survives in that patch. The quality and size of the habitat determines survival of an individual in a previously unoccupied patch [75]. However, assessing habitat quality is more difficult than measuring area of a patch. Hanski [76] argued that the ratio of habitat quality versus area contributions to species occurrence is dependent on certain species-specific factors. For primates, many biogeographic studies found that area was the largest predictor of primate species occurrence [9,10,22,77,78]. For example, Lawes et al. [22] found that area, rather than isolation and habitat disturbance, was the only factor that impacted occurrence in *C*. *m*. *labiatus* in a fragmented landscape. We need future research to evaluate the relative contribution of habitat quality versus area to lemur species occurrence.

#### Connectivity/Isolation

The metapopulation model predicts that connectivity should have a significant positive effect on lemur species occurrence. *E*. *fulvus* was the only species to show a positive relationship in occurrence probability and connectivity. Contrary to model predictions, connectivity had no significant effect on occurrence probability for *C*. *medius* and a negative effect on occurrence probability for *M*. *murinus* and *M*. *ravelobensis*. Migration between fragments is risky for arboreal lemurs. For example, species travelling through the matrix have increased predation risk [71] and there is the possibility of arriving at an unsuitable fragment requiring further migration. In our study, *E*. *fulvus*, although capable of migrating to any fragment and using matrix elements between fragments, appears to stay within the largest and most connected fragments. Although occurring in multiple fragments, *C*. *medius* tended to occur near the largest fragment (Fragment 3 and S2 Table) or the continuous forest. Therefore, they are either not limited by dispersal in a fragmented landscape or they form an intermediate metapopulation (combination of different metapopulations e.g. among patch and mainland-island metapopulations). If *C*. *medius* do represent an intermediate metapopulation, then they are likely to be able to move between fragments but one or more of the larger fragments would act as a mainland source of more colonists [76]. For example, if Fragment 3 acted as a second mainland source, this pattern would explain a lack of difference in occurrence probability between more and less connected fragments. For both *Microcebus* species, the differences in occurrence probability were negative, meaning that the probability of occurrence was lower in closer than in further fragments. One explanation for the negative relationship with connectivity is that the two species of *Microcebus* have higher occurrence probability in more isolated fragments because they are not area limited and are able to survive, possibly in the long-term, within the smallest fragments regardless of isolation. Other studies have recorded *Microcebus* species in all but the smallest (<1 ha) fragments [79–81]. However, these studies did not include as many small (<1 ha) fragments as our study. We found *Microcebus* to occur in fragments as small as 0.23 ha. It is possible that there are source-sink dynamics occurring within the study landscape, where large patches and possibly the nearby continuous forest provide a constant source of potential immigrants for smaller patches [30]. For example, we only observed one *Microcebus* individual in the smallest fragment as well as numerous sightings of mouse lemurs within matrix elements between fragments (Steffens unpublished data), suggesting that occupancy in this patch is ephemeral and thus maintained through colonization via the matrix. Ganzhorn and Schmid [82] also found a potential source-sink relationship occurring for *M*. *murinus* in secondary forests within a fragmented landscape. They observed poorer conditions (smaller, fewer trees and warmer temperatures) within the secondary versus primary forest and within the secondary forest they never re-captured any individuals but were able to recapture seven within the continuous forest.

### Question 3: Within simulated metapopulations over time, how do area and connectivity/isolation affect occurrence?

One of the advantages of a metapopulation approach is that it is possible to model extinction (area) and colonization (connectivity/isolation) probabilities, which allows for the simulation of their effects on occurrence over time. Our simulation results confirm that area is consistently positively related to lemur species occurrence. The simulations on removing the most connected/closest or least connected/furthest may help us understand *Microcebus spp*. metapopulation dynamics within the landscape. *M*. *ravelobensis* occurrence declined when the closest fragments were removed but *M*. *murinus* occurrence did not. This result supports our previous suggestion that *M*. *ravelobensis* is more dispersal limited. They appear to need the closer fragments as stepping-stones to the other fragments whereas *M*. *murinus* isn’t impacted by the proximity of the closest fragments due to their greater dispersal ability.

### Question 4: What are the conservation implications of our findings?

Visual inspection of satellite imagery from 1984 to 2017 [7] shows that human activity has maintained levels of habitat fragmentation in our study site with little to no forest recovery. However, in the same time period forest along the periphery of Ankarafantsika National Park has declined from human activity. The results of our study suggest that maintaining or increasing fragment area within the study site will have the greatest positive benefit to the most lemur species. All analyses suggest that large fragments are crucial to maintaining lemur species metapopulations. Landscape connectivity should be an important variable for each lemur species but our results show that only *E*. *fulvus* occurrence was positively related to connectivity. Connectivity was negatively related to occurrence probability of *M*. *murinus* and *M*. *ravelobensis* and was not significantly related to occurrence probability of *C*. *medius*. To have the maximum benefit for the most species in the short-term we recommend that conservation efforts focus on maintaining or increasing fragment size rather than increasing fragment connectivity. Additionally, increasing fragment size will have the secondary benefit of improving fragment connectivity as the distance between fragments will decrease as fragment size increases. However, we do recommend improving fragment connectivity for conservation measures focusing on *E*. *fulvus* as it is an important seed disperser in the landscape [83] and we found their occurrence to be positively related to fragment connectivity. If source-sink dynamics are occurring within the landscape for *M*. *murinus* and *M*. *ravelobensis*, then we also specifically recommend increasing the area of potential sink fragments to reduce the likely mortality of individuals moving from source populations in large fragments.

## Conclusion

A metapopulation approach is useful for determining the combined and separate effects of area and connectivity/isolation on lemur species occurrence in a fragmented landscape. This study shows that four lemur species (*C*. *medius*, *M*. *ravelobensis*, *M*. *murinus*, and *E*. *fulvus*) form metapopulations in fragmented landscapes. Within their metapopulations, lemurs are impacted by both habitat area and isolation but our study shows that area strongly affects species occurrence and that isolation has species-specific neutral, negative, and positive impacts on lemur species occurrence. We identified dispersal ability and edge tolerance as potentially explaining differences in metapopulation dynamics. It is possible that source-sink dynamics at the studied scale are impacting populations of *M*. *murinus* and *M*. *ravelobensis* within the landscape. The two most frugivorous lemurs, *E*. *fulvus* and *C*. *medius*, may be able to maintain stable metapopulations likely through dispersal and their ability to survive within the largest fragments. To maintain metapopulations for each species, we recommend strategies that reduce further habitat loss and isolation among fragments in the landscape. We should pay special attention to connecting the largest fragments allowing seed dispersers, such as *E*. *fulvus* and *C*. *medius* to increase the area of potential seed deposition.

## Supporting information

S1 Table Transect length and survey data of 42 fragments in a 3,000 ha fragmented landscape. The number of surveys conducted uring the day (diurnal), night (nocturnal), and total. And the number of associated sightings of all lemur species.
(DOCX)

S2 Table Lemur species occurrence. DCF represents distance to continuous forests; CM represents *Cheirogaleus medius* occurrence; MM represents *Microcebus murinus* occurrence; MR represents *Microcebus ravelobensis* occurrence; PC represents *Propithecus coquereli* occurrence; EF represents *Eulemur fulvus* occurrence; LE represents *Lepilemur edwardsi* occurrence.
(DOCX)
