# Supplementary material for: Lemur species-specific metapopulation responses to habitat loss and fragmentation
Source: PLoS One. 2018 May 9;13(5):e0195791. doi: 10.1371/journal.pone.0195791 (PMC5942715; doi:10.1371/journal.pone.0195791)
Supplement: S1 Table — The number of surveys conducted uring the day (diurnal), night (nocturnal), and total. And the number of associated sightings of all lemur species. (DOCX) [file pone.0195791.s001.docx]

**S1. Transect Length and Survey Data of 42 Fragments in a 3,000 ha Fragmented Landscape.**

| Fragment ID | Transect Length (m)* | Diurnal Surveys | Nocturnal Surveys | Total Surveys |  | CM | MM | MR | Mspp. | PC | EF | LE | **Total** |
| --- | --- | --- | --- | --- | --- | --- | --- | --- | --- | --- | --- | --- | --- |
| 1 | 823.1 | 15 | 15 | 30 | Diurnal | 0 | 0 | 0 | 0 | 0 | 1 | 0 | **1** |
|  |  |  |  |  | Nocturnal | 1 | 10 | 20 | 15 | 0 | 0 | 0 | **46** |
| 2 | 1629.9 | 15 | 15 | 30 | Diurnal | 0 | 0 | 0 | 0 | 1 | 3 | 0 | **4** |
|  |  |  |  |  | Nocturnal | 2 | 31 | 45 | 51 | 0 | 3 | 0 | **132** |
| 3 | 1804.2 | 18 | 21 | 39 | Diurnal | 0 | 0 | 0 | 0 | 8 | 10 | 0 | **18** |
|  |  |  |  |  | Nocturnal | 6 | 23 | 40 | 62 | 3 | 12 | 5 | **151** |
| 4 | 565.8 | 14 | 18 | 32 | Diurnal | 0 | 0 | 0 | 0 | 4 | 2 | 0 | **6** |
|  |  |  |  |  | Nocturnal | 0 | 12 | 12 | 13 | 2 | 2 | 0 | **41** |
| 5 | 627.3 | 15 | 15 | 30 | Diurnal | 0 | 0 | 0 | 0 | 0 | 2 | 0 | **2** |
|  |  |  |  |  | Nocturnal | 1 | 20 | 12 | 25 | 0 | 2 | 0 | **60** |
| 6 | 649.1 | 15 | 17 | 32 | Diurnal | 0 | 0 | 0 | 0 | 0 | 0 | 0 | **0** |
|  |  |  |  |  | Nocturnal | 2 | 20 | 8 | 24 | 0 | 0 | 1 | **55** |
| 7 | 278 | 14 | 17 | 31 | Diurnal | 0 | 0 | 0 | 0 | 0 | 0 | 0 | **0** |
|  |  |  |  |  | Nocturnal | 1 | 0 | 4 | 5 | 0 | 1 | 0 | **11** |
| 8 | 621.3 | 14 | 17 | 31 | Diurnal | 0 | 0 | 0 | 0 | 0 | 1 | 0 | **1** |
|  |  |  |  |  | Nocturnal | 0 | 11 | 4 | 14 | 0 | 1 | 0 | **30** |
| 9 | 625.4 | 15 | 17 | 32 | Diurnal | 0 | 0 | 0 | 0 | 0 | 0 | 0 | **0** |
|  |  |  |  |  | Nocturnal | 2 | 14 | 17 | 13 | 0 | 0 | 0 | **46** |
| 10 | 294.6 | 14 | 14 | 28 | Diurnal | 0 | 0 | 0 | 0 | 0 | 0 | 0 | **0** |
|  |  |  |  |  | Nocturnal | 0 | 11 | 5 | 15 | 0 | 0 | 0 | **31** |
| 11 | 227.3 | 14 | 13 | 27 | Diurnal | 0 | 0 | 0 | 0 | 0 | 0 | 0 | **0** |
|  |  |  |  |  | Nocturnal | 0 | 1 | 5 | 2 | 0 | 0 | 0 | **8** |
| 12a | 430.7 | 14 | 13 | 27 | Diurnal | 0 | 0 | 0 | 0 | 0 | 0 | 0 | **0** |
|  |  |  |  |  | Nocturnal | 0 | 8 | 10 | 7 | 0 | 0 | 0 | **25** |
| 12b | 577 | 13 | 13 | 26 | Diurnal | 0 | 0 | 0 | 0 | 0 | 0 | 0 | **0** |
|  |  |  |  |  | Nocturnal | 1 | 3 | 5 | 6 | 0 | 0 | 0 | **15** |
| 13 | 314 | 14 | 13 | 27 | Diurnal | 0 | 0 | 0 | 0 | 0 | 0 | 0 | **0** |
|  |  |  |  |  | Nocturnal | 0 | 5 | 6 | 8 | 0 | 0 | 0 | **19** |
| 14 | 291.3 | 14 | 16 | 30 | Diurnal | 0 | 0 | 0 | 0 | 0 | 0 | 0 | **0** |
|  |  |  |  |  | Nocturnal | 0 | 7 | 2 | 6 | 0 | 0 | 0 | **15** |
| 15 | 148.6 | 16 | 16 | 32 | Diurnal | 0 | 0 | 0 | 0 | 0 | 0 | 0 | **0** |
|  |  |  |  |  | Nocturnal | 0 | 6 | 7 | 11 | 0 | 0 | 0 | **24** |
| 16 | 105 | 14 | 16 | 30 | Diurnal | 0 | 0 | 0 | 0 | 0 | 0 | 0 | **0** |
|  |  |  |  |  | Nocturnal | 0 | 0 | 1 | 1 | 0 | 0 | 0 | **2** |
| 17 | 66.3 | 14 | 16 | 30 | Diurnal | 0 | 0 | 0 | 0 | 0 | 0 | 0 | **0** |
|  |  |  |  |  | Nocturnal | 0 | 0 | 0 | 0 | 0 | 0 | 0 | **0** |
| 18 | 289.5 | 13 | 16 | 29 | Diurnal | 0 | 0 | 0 | 0 | 0 | 0 | 0 | **0** |
|  |  |  |  |  | Nocturnal | 0 | 1 | 0 | 3 | 0 | 0 | 0 | **4** |
| 19 | 210 | 13 | 16 | 29 | Diurnal | 0 | 0 | 0 | 0 | 0 | 0 | 0 | **0** |
|  |  |  |  |  | Nocturnal | 0 | 2 | 1 | 4 | 0 | 0 | 0 | **7** |
| 20 | 161.8 | 14 | 16 | 30 | Diurnal | 0 | 0 | 0 | 0 | 0 | 0 | 0 | **0** |
|  |  |  |  |  | Nocturnal | 0 | 10 | 7 | 3 | 0 | 0 | 0 | **20** |
| 21 | 67.7 | 14 | 16 | 30 | Diurnal | 0 | 0 | 0 | 0 | 0 | 0 | 0 | **0** |
|  |  |  |  |  | Nocturnal | 0 | 0 | 0 | 0 | 0 | 0 | 0 | **0** |
| 22 | 43.8 | 14 | 16 | 30 | Diurnal | 0 | 0 | 0 | 0 | 0 | 0 | 0 | **0** |
|  |  |  |  |  | Nocturnal | 0 | 1 | 1 | 2 | 0 | 0 | 0 | **4** |
| 23 | 325.2 | 15 | 15 | 30 | Diurnal | 0 | 0 | 0 | 0 | 0 | 0 | 0 | **0** |
|  |  |  |  |  | Nocturnal | 0 | 7 | 4 | 6 | 0 | 0 | 0 | **17** |
| 24 | 61.1 | 14 | 14 | 28 | Diurnal | 0 | 0 | 0 | 0 | 0 | 0 | 0 | **0** |
|  |  |  |  |  | Nocturnal | 0 | 1 | 2 | 2 | 0 | 0 | 0 | **5** |
| 25 | 86.7 | 16 | 15 | 31 | Diurnal | 0 | 0 | 0 | 0 | 0 | 0 | 0 | **0** |
|  |  |  |  |  | Nocturnal | 0 | 0 | 2 | 5 | 0 | 0 | 0 | **7** |
| 27 | 188.3 | 14 | 14 | 28 | Diurnal | 0 | 0 | 0 | 0 | 0 | 0 | 0 | **0** |
|  |  |  |  |  | Nocturnal | 0 | 2 | 0 | 2 | 0 | 0 | 0 | **4** |
| 28 | 76.8 | 15 | 13 | 28 | Diurnal | 0 | 0 | 0 | 0 | 0 | 0 | 0 | **0** |
|  |  |  |  |  | Nocturnal | 0 | 4 | 1 | 1 | 0 | 0 | 0 | **6** |
| 29 | 138.3 | 14 | 15 | 29 | Diurnal | 0 | 0 | 0 | 0 | 0 | 0 | 0 | **0** |
|  |  |  |  |  | Nocturnal | 0 | 1 | 0 | 0 | 0 | 0 | 0 | **1** |
| 30 | 119.1 | 15 | 14 | 29 | Diurnal | 0 | 0 | 0 | 0 | 0 | 0 | 0 | **0** |
|  |  |  |  |  | Nocturnal | 0 | 0 | 0 | 0 | 0 | 0 | 0 | **0** |
| 31 | 535.6 | 14 | 14 | 28 | Diurnal | 0 | 0 | 0 | 0 | 0 | 0 | 0 | **0** |
|  |  |  |  |  | Nocturnal | 3 | 4 | 9 | 5 | 0 | 0 | 0 | **21** |
| 32 | 239.8 | 14 | 14 | 28 | Diurnal | 0 | 0 | 0 | 0 | 0 | 0 | 0 | **0** |
|  |  |  |  |  | Nocturnal | 0 | 11 | 4 | 2 | 0 | 0 | 0 | **17** |
| 33 | 324.7 | 14 | 14 | 28 | Diurnal | 0 | 0 | 2 | 0 | 0 | 0 | 0 | **2** |
|  |  |  |  |  | Nocturnal | 0 | 4 | 1 | 2 | 0 | 0 | 0 | **7** |
| 34 | 278.2 | 13 | 14 | 27 | Diurnal | 0 | 0 | 0 | 0 | 0 | 0 | 0 | **0** |
|  |  |  |  |  | Nocturnal | 2 | 9 | 14 | 10 | 0 | 0 | 0 | **35** |
| 35 | 377.1 | 15 | 14 | 29 | Diurnal | 0 | 0 | 0 | 0 | 0 | 0 | 0 | **0** |
|  |  |  |  |  | Nocturnal | 0 | 1 | 1 | 9 | 0 | 0 | 0 | **11** |
| 36 | 314.2 | 14 | 14 | 28 | Diurnal | 0 | 0 | 0 | 0 | 0 | 0 | 0 | **0** |
|  |  |  |  |  | Nocturnal | 1 | 7 | 8 | 8 | 0 | 0 | 0 | **24** |
| 37 | 127.5 | 14 | 14 | 28 | Diurnal | 0 | 0 | 0 | 0 | 0 | 0 | 0 | **0** |
|  |  |  |  |  | Nocturnal | 0 | 0 | 0 | 0 | 0 | 0 | 0 | **0** |
| 38 | 178.3 | 14 | 14 | 28 | Diurnal | 0 | 0 | 0 | 0 | 0 | 0 | 0 | **0** |
|  |  |  |  |  | Nocturnal | 0 | 1 | 0 | 1 | 0 | 0 | 0 | **2** |
| 39 | 498.2 | 14 | 13 | 27 | Diurnal | 0 | 0 | 0 | 0 | 0 | 0 | 0 | **0** |
|  |  |  |  |  | Nocturnal | 0 | 14 | 8 | 19 | 0 | 0 | 0 | **41** |
| 40 | 391 | 14 | 13 | 27 | Diurnal | 0 | 0 | 0 | 0 | 0 | 0 | 0 | **0** |
|  |  |  |  |  | Nocturnal | 0 | 5 | 6 | 11 | 0 | 0 | 0 | **22** |
| 41 | 223 | 11 | 11 | 22 | Diurnal | 0 | 0 | 0 | 0 | 0 | 0 | 0 | **0** |
|  |  |  |  |  | Nocturnal | 0 | 3 | 4 | 5 | 0 | 0 | 0 | **12** |
| 42 | 141.9 | 12 | 11 | 23 | Diurnal | 0 | 0 | 0 | 0 | 0 | 0 | 0 | **0** |
|  |  |  |  |  | Nocturnal | 0 | 0 | 9 | 4 | 0 | 0 | 0 | **13** |
| **Total** | **15476.7** | **596** | **622** | **1218** |  | **22** | **270** | **287** | **382** | **18** | **40** | **6** | **1025** |

The number of surveys conducted during the day (diurnal), night (nocturnal), and total and the number of associated sightings by species. There was one transect per fragment accept fragment 3 which had two transects. CM represents *Chreirgaleus medius*, MM represents *Mircrocebus murinus*, MR represents *Microcebus ravelobensis*, Mspp. represents *Microcebus species* together, PC represents *Propithecus coquereli*, EF represents *Eulemur fulvus*, LE represents *Lepilemur edwardsi*.
