# Supplementary material for: Lemur species-specific metapopulation responses to habitat loss and fragmentation
Source: PLoS One. 2018 May 9;13(5):e0195791. doi: 10.1371/journal.pone.0195791 (PMC5942715; doi:10.1371/journal.pone.0195791)
Supplement: S2 Table — DCF represents distance to continuous forests; CM represents Cheirogaleus medius occurrence; MM represents Microcebus murinus occurrence; MR represents Microcebus ravelobensis occurrence; PC represents Propithecus coquereli occurrence; EF represents Eulemur fulvus occurrence; LE represents Lepilemur edwardsi occurrence. (DOCX) [file pone.0195791.s002.docx]

**Lemur Species Occurrence.**

| Fragment | Easting | Northing | Area (ha) | DCF (m) | CM | MM | MR | PC | EF | LE |
| --- | --- | --- | --- | --- | --- | --- | --- | --- | --- | --- |
| 1 | 681229.5354 | 8196249.23 | 34.51 | 2.1986 | 1 | 1 | 1 | 0 | 1 | 0 |
| 2 | 683046.3204 | 8196770.685 | 45.34 | 0.6361 | 1 | 1 | 1 | 1 | 1 | 0 |
| 3 | 680929.4501 | 8194782.459 | 117.70 | 0.8190 | 1 | 1 | 1 | 1 | 1 | 1 |
| 4 | 682166.8703 | 8195602.191 | 19.46 | 1.3200 | 0 | 1 | 1 | 1 | 1 | 0 |
| 5 | 680367.0489 | 8194622.587 | 16.01 | 1.1532 | 1 | 1 | 1 | 0 | 1 | 0 |
| 6 | 683221.3463 | 8194719.551 | 11.58 | 0.1797 | 1 | 1 | 1 | 0 | 0 | 1 |
| 7 | 681144.3724 | 8193875.477 | 4.16 | 1.3857 | 1 | 0 | 1 | 0 | 1 | 0 |
| 8 | 681599.3127 | 8193992.831 | 13.55 | 1.1546 | 1 | 1 | 1 | 0 | 1 | 0 |
| 9 | 682975.0209 | 8194096.689 | 15.38 | 0.1108 | 1 | 1 | 1 | 0 | 0 | 0 |
| 10 | 682743.6007 | 8192615.995 | 2.78 | 0.1059 | 0 | 1 | 1 | 0 | 0 | 0 |
| 11 | 683201.2339 | 8198115.78 | 2.57 | 0.2101 | 0 | 1 | 1 | 0 | 0 | 0 |
| 12a | 683926.0568 | 8196816.704 | 14.22 | 0.6563 | 0 | 1 | 1 | 0 | 0 | 0 |
| 12b | 683465.507 | 8196450.35 | 5.18 | 0.8424 | 1 | 1 | 1 | 0 | 0 | 0 |
| 13 | 684967.5505 | 8197135.806 | 3.75 | 0.2777 | 0 | 1 | 1 | 0 | 0 | 0 |
| 14 | 680528.4188 | 8195689.291 | 4.08 | 2.0089 | 0 | 1 | 1 | 0 | 0 | 0 |
| 15 | 681346.6743 | 8195999.279 | 1.18 | 2.4814 | 0 | 1 | 1 | 0 | 0 | 0 |
| 16 | 681764.8952 | 8195853.506 | 0.76 | 2.0446 | 0 | 0 | 1 | 0 | 0 | 0 |
| 17 | 681692.4645 | 8195078.336 | 0.42 | 1.8318 | 0 | 0 | 0 | 0 | 0 | 0 |
| 18 | 681989.5223 | 8194245.285 | 1.14 | 1.0587 | 0 | 1 | 0 | 0 | 0 | 0 |
| 19 | 682272.137 | 8194521.243 | 1.40 | 1.0885 | 0 | 1 | 1 | 0 | 0 | 0 |
| 20 | 683540.3321 | 8195229.336 | 1.43 | 0.1805 | 0 | 1 | 1 | 0 | 0 | 0 |
| 21 | 681271 | 8193430.4 | 0.38 | 1.0909 | 0 | 0 | 0 | 0 | 0 | 0 |
| 22 | 682997.3183 | 8193680.934 | 0.23 | 0.1037 | 0 | 1 | 1 | 0 | 0 | 0 |
| 23 | 680334.2891 | 8196709.221 | 2.48 | 2.0516 | 0 | 1 | 1 | 0 | 0 | 0 |
| 24 | 682343.8088 | 8193354.35 | 0.28 | 0.4523 | 0 | 1 | 1 | 0 | 0 | 0 |
| 25 | 680484.0239 | 8193698.671 | 0.57 | 0.9926 | 0 | 0 | 1 | 0 | 0 | 0 |
| 27 | 680447.6819 | 8193128.557 | 1.97 | 0.7475 | 0 | 1 | 0 | 0 | 0 | 0 |
| 28 | 680025.7513 | 8195775.818 | 0.64 | 1.9298 | 0 | 1 | 1 | 0 | 0 | 0 |
| 29 | 680025.1077 | 8195927.361 | 0.71 | 2.0748 | 0 | 1 | 0 | 0 | 0 | 0 |
| 30 | 680211.8322 | 8196333.512 | 0.31 | 2.4783 | 0 | 0 | 0 | 0 | 0 | 0 |
| 31 | 680574.6924 | 8197350.687 | 17.03 | 1.1869 | 1 | 1 | 1 | 0 | 0 | 0 |
| 32 | 680694.836 | 8197913.172 | 0.98 | 0.8037 | 0 | 1 | 1 | 0 | 0 | 0 |
| 33 | 680782.7196 | 8198237.823 | 2.16 | 0.4331 | 0 | 1 | 1 | 0 | 0 | 0 |
| 34 | 680365.9717 | 8198641.712 | 1.69 | 0.2683 | 1 | 1 | 1 | 0 | 0 | 0 |
| 35 | 681473.8008 | 8198372.809 | 5.00 | 0.4137 | 0 | 1 | 1 | 0 | 0 | 0 |
| 36 | 681893.4989 | 8197341.167 | 6.97 | 1.3360 | 1 | 1 | 1 | 0 | 0 | 0 |
| 37 | 682753.1911 | 8197404.389 | 0.64 | 0.7880 | 0 | 0 | 0 | 0 | 0 | 0 |
| 38 | 681946.3878 | 8197648.017 | 0.52 | 1.1821 | 0 | 1 | 0 | 0 | 0 | 0 |
| 39 | 682820.8662 | 8198394.743 | 5.17 | 0.3216 | 0 | 1 | 1 | 0 | 0 | 0 |
| 40 | 683355.6463 | 8198901.49 | 3.78 | 0.0951 | 0 | 1 | 1 | 0 | 0 | 0 |
| 41 | 683518.9299 | 8198737.51 | 1.56 | 0.0171 | 0 | 1 | 1 | 0 | 0 | 0 |
| 42 | 682903.0047 | 8198622.145 | 0.79 | 0.2633 | 0 | 1 | 1 | 0 | 0 | 0 |

DCF represents distance to continuous forests; CM represents *Cheirogaleus medius* occurrence; MM represents *Microcebus murinus* occurrence; MR represents *Microcebus ravelobensis* occurrence; PC represents *Propithecus coquereli* occurrence; EF represents *Eulemur fulvus* occurrence; LE represents *Lepilemur edwardsi* occurrence.
